# Biomechanical simulation of vocal fold dynamics in adults based on laryngeal high-speed videoendoscopy

**DOI:** 10.1371/journal.pone.0187486

**Published:** 2017-11-09

**Authors:** Michael Döllinger, Pablo Gómez, Rita R. Patel, Christoph Alexiou, Christopher Bohr, Anne Schützenberger

**Affiliations:** 1 Division of Phoniatrics and Pediatric Audiology, Department of Otorhinolaryngology, Head and Neck Surgery, Medical School, Friedrich-Alexander-University Erlangen-Nürnberg, Erlangen, Germany; 2 Department of Speech and Hearing Sciences, Indiana University, Bloomington, Indiana, Indiana, United States of America; 3 Section of Experimental Oncology and Nanomedicine (SEON), Department of Otorhinolaryngology, Head and Neck Surgery, Medical School, Else Kröner-Fresenius-Stiftung-Professorship, Friedrich-Alexander-University Erlangen-Nürnberg, Erlangen, Germany; University of Waterloo, CANADA

## Abstract

**Motivation:**

Human voice is generated in the larynx by the two oscillating vocal folds. Owing to the limited space and accessibility of the larynx, endoscopic investigation of the actual phonatory process in detail is challenging. Hence the biomechanics of the human phonatory process are still not yet fully understood. Therefore, we adapt a mathematical model of the vocal folds towards vocal fold oscillations to quantify gender and age related differences expressed by computed biomechanical model parameters.

**Methods:**

The vocal fold dynamics are visualized by laryngeal high-speed videoendoscopy (4000 fps). A total of 33 healthy young subjects (16 females, 17 males) and 11 elderly subjects (5 females, 6 males) were recorded. A numerical two-mass model is adapted to the recorded vocal fold oscillations by varying model masses, stiffness and subglottal pressure. For adapting the model towards the recorded vocal fold dynamics, three different optimization algorithms (Nelder–Mead, Particle Swarm Optimization and Simulated Bee Colony) in combination with three cost functions were considered for applicability. Gender differences and age-related kinematic differences reflected by the model parameters were analyzed.

**Results and conclusion:**

The biomechanical model in combination with numerical optimization techniques allowed phonatory behavior to be simulated and laryngeal parameters involved to be quantified. All three optimization algorithms showed promising results. However, only one cost function seems to be suitable for this optimization task. The gained model parameters reflect the phonatory biomechanics for men and women well and show quantitative age- and gender-specific differences. The model parameters for younger females and males showed lower subglottal pressures, lower stiffness and higher masses than the corresponding elderly groups. Females exhibited higher subglottal pressures, smaller oscillation masses and larger stiffness than the corresponding similar aged male groups.

Optimizing numerical models towards vocal fold oscillations is useful to identify underlying laryngeal components controlling the phonatory process.

## Introduction

**Fig 1:**
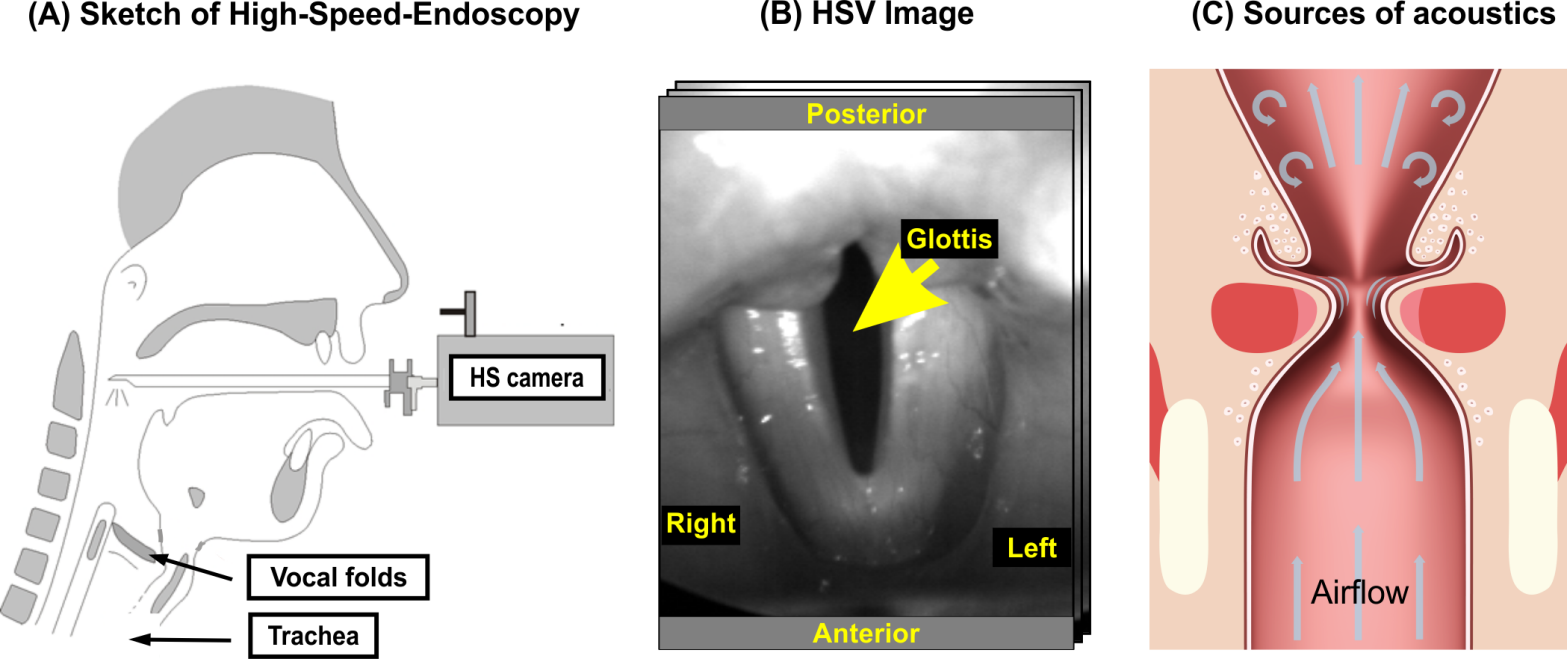
(A) Sketch of the sagittal section of the head and neck, indicating the vocal folds and the rigid endoscope. (B) Glottis (dark) and on the left and right sides the two vocal folds as seen through the HSV. (C) Larynx with highlighted acoustic sound sources (arrows) resulting from the vocal fold vibrations.

The human voice represents an essential aspect of oral communication between human beings. Voice is formed by the interaction and coordination of applied air flow, vocal fold tissue and vocal fold movements. Accurate and precise physiologic interaction of several laryngeal muscle group movements is the basis for normal voice production [1]. The acoustic voice signal originates in the larynx where the two opposing vocal folds are excited by an airflow generated by the lungs (Fig 1). When starting the voice production process (i.e., phonation), the vocal folds are positioned close to each other. Airflow produced from the lungs streams upwards and increases the subglottal pressure below the vocal folds. After exceeding a certain subglottal pressure level, the vocal folds first start to produce small oscillatory motions that then result in a steady-state oscillation (i.e., periodic opening and closing of the vocal folds). A healthy voice signal is normally produced by periodic and symmetric vocal fold oscillations. Also, the closure of the glottis, where the vocal folds almost or entirely close, is considered to be an important part of the normal phonation process [2].

Depending on gender and age, vocal folds oscillate between approximately 100 and 350 times per second during normal phonation [3]: women (~ 200 Hz– 250 Hz), men (~ 100 Hz– 150 Hz), children (~ 200 Hz– 350 Hz). As these movements are so fast, vocal fold dynamics are best captured and visualized by laryngeal high-speed videoendoscopy (HSV) with recording frame rates between 4000 Hz and 20 000 Hz [4–8] (Fig 1A).

Since HSV was first introduced, image processing methods have been proposed [9–11] to allow the quantification of vibratory behavior with objectively computed perturbation measures [12–14]. The signals extracted from HSV and analyzed are either the glottal area waveform (i.e., glottis area function over time) or the vocal fold trajectories at a specific vocal fold location, preferably at mid-membranous position [15–17]. Both signals represent the oscillatory behavior of the vocal folds; i.e., the opening and closing process. Quantitative analysis based on HSV has added substantial knowledge regarding normal and pathological vocal fold dynamics [4,18,19].

Vocal fold dynamics are highly sensitive towards anatomic tissue changes [2], dysfunctions of the involved muscles [20] and subglottal air pressure [21]. Alterations of these parameters may yield disturbed dynamics, resulting in hoarseness. Typically disturbed dynamics are left–right asymmetries, aperiodicities or glottis closure insufficiency, where the vocal folds do not entirely close [2]. It is highly desirable to early diagnose and quantify pathologic dynamic laryngeal alterations to prevent severe laryngeal tissue damage [22,23].

However, because of the limited space in the larynx, it is difficult to measure and quantify the mechanical laryngeal tissue characteristics directly in-vivo [24]. Additionally, HSV evaluation alone only allows vocal fold dynamics to be described but does not give quantitative information on biomechanical parameters such as tissue elasticity and occurring subglottal air pressure. Hence indirect analysis methods based on the adaptation of numeric biomechanical larynx models towards in-vivo HSV recorded vocal fold dynamics were suggested [25,26].

**Fig 2:**
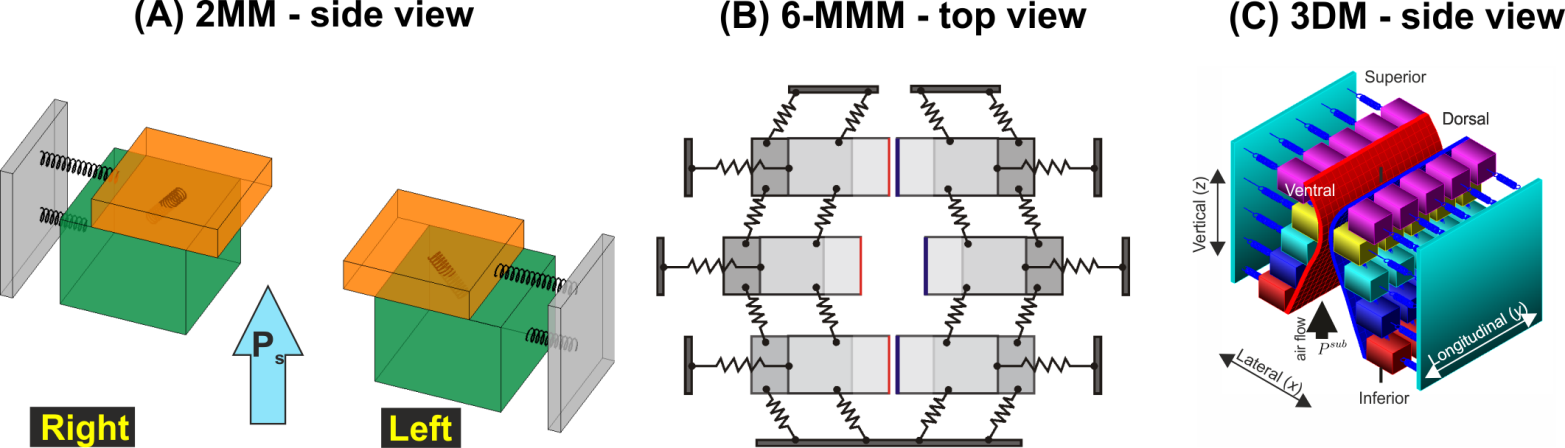
(A) Two-mass model as used in this work with indicated subglottal pressure P_s_. (B) Six-mass model allowing the simulation of the vocal fold trajectories along three positions, i.e. at posterior, medial and anterior positions of the vocal folds. (C) Three-dimensional multi-mass model that additionally allows the simulation of vertical dynamics and the vocal fold medial surface at 25 positions along each vocal fold.

Initially, these biomechanical models were used to simulate the underlying processes during phonation. Models were developed to allow the investigation of parameter effects such as applied subglottal air pressure, vibrating masses, tissue stiffness and elongation characteristics with respect to the dynamic vocal fold behavior [27–29]. These so-called lumped mass models (LMMs) are fairly simple but still enable many dynamic characteristics in the larynx to be reproduced [30]. In the most basic models, the vocal folds are simulated by a self-vibrating source consisting of two spring-coupled masses (2MMs) for each vocal fold (Fig 2A) [31]. Since these 2MMs allowed the simulation of only one trajectory at one vocal fold position, models with more masses (multi-mass models, MMMs) were suggested to permit the simultaneous simulation of the vocal fold dynamics at different positions (Fig 2B) [32]. However, the 2MMs and MMMs only focused on the simulation of the lateral (i.e., horizontal) vocal fold displacements and on the phase differences along the inferior-superior plane. To simulate the often neglected vertical vocal fold movements [33] (i.e., vertical tissue displacement in inferior-superior direction), enhanced and more complex three-dimensional LMMs were introduced [34] (Fig 2C).

After the development of the LMMs to simulate human vocal fold dynamics by manually adapting the model parameter settings [28,35], the automatic optimization of these numerical models towards HSV recorded vocal fold oscillations was suggested in order to acquire information on parameters responsible for chaotic behavior, certain dynamic conditions and left–right asymmetric oscillations.

The first fully automatic optimization method was realized by using the Nelder–Mead algorithm to automatically optimize the parameters of a 2MM to reproduce HSV recorded human vocal fold dynamics during sustained phonation [25,26]. Three parameters (vibrating mass, stiffness and subglottal pressure) were varied in the so called cost function Γ to minimize the involved periodic oscillatory components, represented by discrete Fourier transformation (DFT) coefficients, between the model trajectories and trajectories extracted from HSV recordings. Based on this work, the 2MM and a genetic algorithm for the optimization were successfully employed to reproduce the trajectories of patients suffering from unilateral vocal fold paralysis [36] and for ex-vivo larynx experiments [37]. Combining a genetic algorithm with a quasi-Newton method a 2MM successfully reproduced vocal fold dynamics in three subjects [38]. As cost function the Euclidean distance of the glottal area waveform was chosen. Lately, statistical methods like a non-stationary Bayesian estimation approach were suggested for optimizing 2MMs but was only tested on theoretical (i.e., simulated) vocal fold oscillations [39]. Further, a time-dependent 2MM was successfully adapted for 20 healthy and pathological voices [40]. For the optimization an adaptive Simulated Annealing approach was chosen to minimize the Euclidean distance between the model and experimental vocal fold trajectories.

Schwarz et al (2008) [41] successfully optimized a MMM with six coupled masses (6-MMM) towards six disordered voices and two normal voices. They applied a Genetic algorithm and split the optimization process into several sub-steps. As cost function Γ, they used a combination of glottal area and vocal fold trajectory consistency measure. Also, MMMs were used to reproduce vocal fold dynamics within pitch rise paradigms [42]. Wavelet coefficients in the cost function were chosen to consider the time dependency of the system. Powell’s Direction Set optimization algorithm was applied to the interval divided (i.e., four oscillation cycles) signal. Thereby, 30 healthy and pathological adult voices were adapted.

Simulating the phonatory process after total laryngectomy, due to cancer, was achieved through coupling eight two-mass models arranged in a circle [43]. For the optimization they selected a combination of Simulated Annealing for a preliminary global search and Powell’s Direction Set method for the final local approximation. As cost function a combination of area difference, intersection and distance measures was chosen. The method was tested on 75 synthetic data sets and four human subjects.

Finally, a three-dimensional model (3DM) was developed and applied to ex-vivo human vocal fold dynamics produced in a hemi-larynx setup [44]. This model allowed the simulation of the entire vocal fold surface from inferior to superior including the vertical dynamics [44]. Owing to the increased number of masses in the 3DM (i.e., 25 on each side) and the rather unfortunate topology of the cost function (i.e., many local minima), the optimization was divided into several coarse steps, followed by fine optimization processes. It started with a global optimization combining Particle Swarm Optimization and Simulated Annealing algorithms. The local optimization was achieved by Powell’s direction set method [45]. By adapting this model to ex-vivo human larynx dynamics, information was obtained on the distribution of vibrating masses and stiffness along the vocal fold surface. The optimization results accurately matched the actual fundamental frequencies and also the experimental measured subglottal pressure values [44].

Overviews of current applied LMMs can be found in [46,47]. Owing to the increase in computational power, more accurate and therefore more computationally intensive so called Finite Element Methods (FEM) and Finite Volume Methods (FVM) simulating 2D and 3D laryngeal dynamics and airflow became popular during the last decade [31,48–50]. However, the complexity of these models and therefore the computational costs do not allow them to be used for optimization purposes yet.

As described above, numerical optimization of LMMs has so far focused only on healthy adults and compared model parameters for healthy vs. disordered phonation processes.

To the best of our knowledge no studies have applied LMMs to analyze gender specific differences. Previous ex-vivo and in-vivo studies on human larynges showed the following, partly contradictory, results: Regarding vocal fold stiffness, an ex-vivo study reported, at medial position slightly above the vocal fold edge, smaller stiffness for an elderly women compared to an elderly male [51]. In contrast, it was suggested that vocal folds in men are less stiff than in women [52]; unfortunately the authors did not report the age of the subjects. Gender related subglottal pressure differences were reported for young adults [53]; another study, for example showed no gender specific differences [54, 55]. However, male vocal folds are known to be larger (i.e., increased mass) than female vocal folds [56].

Further, to the best of our knowledge no studies have compared biomechanical LMM parameters between younger and elderly subjects. Previous ex-vivo and in-vivo studies on human larynges reported the following results: Glottal parameters extracted from HSV recordings were successfully applied to differentiate age groups; however no details on the parameters quantities were presented [57]. Age-related morphological changes of the vocal folds influencing the viscoelasticity have been described [58–61]. Previous studies suggested lower stiffness for younger males compared to elderly males [51,62]. Histologic analysis reported reduced lamina propria thickness and reduced epithelial cell density for elderly subjects [63]. However, an increase in vocal fold volume (i.e., mass) for 28.7% of analyzed elderly women was also reported [62]. Furthermore, a thickening of mucosa and vocal fold cover in elderly women was described [64]. Higher subglottal pressure in elderly males compared to younger male subjects was suggested [65]. However, other studies reported no differences [66,67]. It was also suggested that aging effects on phonatory behaviors differ in degree and kind for men and women [65]. In summary, a better understanding of the elderly voice and accompanying voice disorders is desired [66,68].

Since it is also not obvious, which optimization algorithms and cost functions are the most appropriate ones, we have decided to apply different approaches in our study. Therefore, by optimizing a LMM towards vocal fold oscillations recorded by endoscopic high-speed imaging, the aims of our study are:

1. to analyze the performance of three different optimization algorithms and three different cost functions (Γ_1_, Γ_2_, Γ_3_) to automatically adapt a fairly simple LMM (i.e., in our study 2MM) to vocal fold dynamics.
2. to quantify gender related biomechanical differences in the larynx expressed by 2MM parameters. We hypothesize that healthy young males would have greater masses, lower stiffness and less subglottal pressure compared to healthy young females.
3. to quantify age related biomechanical differences in the larynx expressed by 2MM parameters. We hypothesize that healthy young adults would have greater vocal fold masses, lower vocal fold stiffness and lower subglottal pressure compared to elderly atrophic subjects. Also, it is expected to find higher kinematic asymmetry in the elderly subjects as reported previously [69,70].

By investigating these objectives, we want to illustrate and emphasize the informative value of LMMs towards vocal fold physiology by analyzing interrelations between underlying laryngeal components such as vibrating mass, stiffness and applied subglottal pressure. Further, we want to illustrate the potential for the differentiation of vocal fold vibratory characteristics based on LMMs.

## Methods

### Subjects

Four groups of subjects were investigated. Two younger age groups were analyzed with 16 healthy females (18–24 years) and 17 healthy males (19–38 years). The subjects were recruited at the FAU-Erlangen-Nürnberg and University of Kentucky. The subjects had to fulfill the following criteria: negative history of vocal pathology, not a professional vocal user and having a normal voice, as confirmed by a speech–language pathologist. Adults with history of smoking were excluded.

Further six older male subjects (62–86 years) and five older female subjects (72–91 years) were analyzed. These subjects were recruited during regular office hours at the Division of Phoniatrics and Pediatric Audiology of the department Otolaryngology, Head- and Neck surgery at the University Hospital Erlangen. The subjects were diagnosed with vocal fold atrophy and had no further vocal fold pathologies.

The participants gave informed, written consent prior to the participation and this consent procedure was approved by the corresponding local ethics committees (Ethik-Kommission der Medizinischen Fakultät FAU-Erlangen-Nürnberg and Office of Research Integrity Expedited Review Board at the University of Kentucky). Experiments were performed in accordance with the Declaration of Helsinki (1964).

### Data acquisition

To record the vocal fold vibrations, a PENTAX Medical (Montvale, NJ, USA) Model 9710 digital gray scale high-speed (HS) camera was used at both universities. The applied temporal resolution of 4000 fps enables the vocal fold oscillations to be captured [4]. The HS camera provides a series of pictures with an image resolution of 512 × 256 pixels with a maximum duration of 4 seconds to visualize the laryngeal dynamics.

The recordings were performed during sustained phonation of the vowel /i:/ with a PENTAX Medical 70° endoscope containing a 300 Watt Xenon light source. For each subject, one sample of typical phonation was recorded and analyzed. For the optimization, a sequence length of 100 ms (N = 400 frames) of sustained phonation was chosen. This interval covered between 10 and 50 oscillation cycles depending on the fundamental frequency. The choice of this interval length is a compromise between a sufficient observation time and computational costs and lies in the range of previous reported interval lengths.

### Data processing

**Fig 3:**
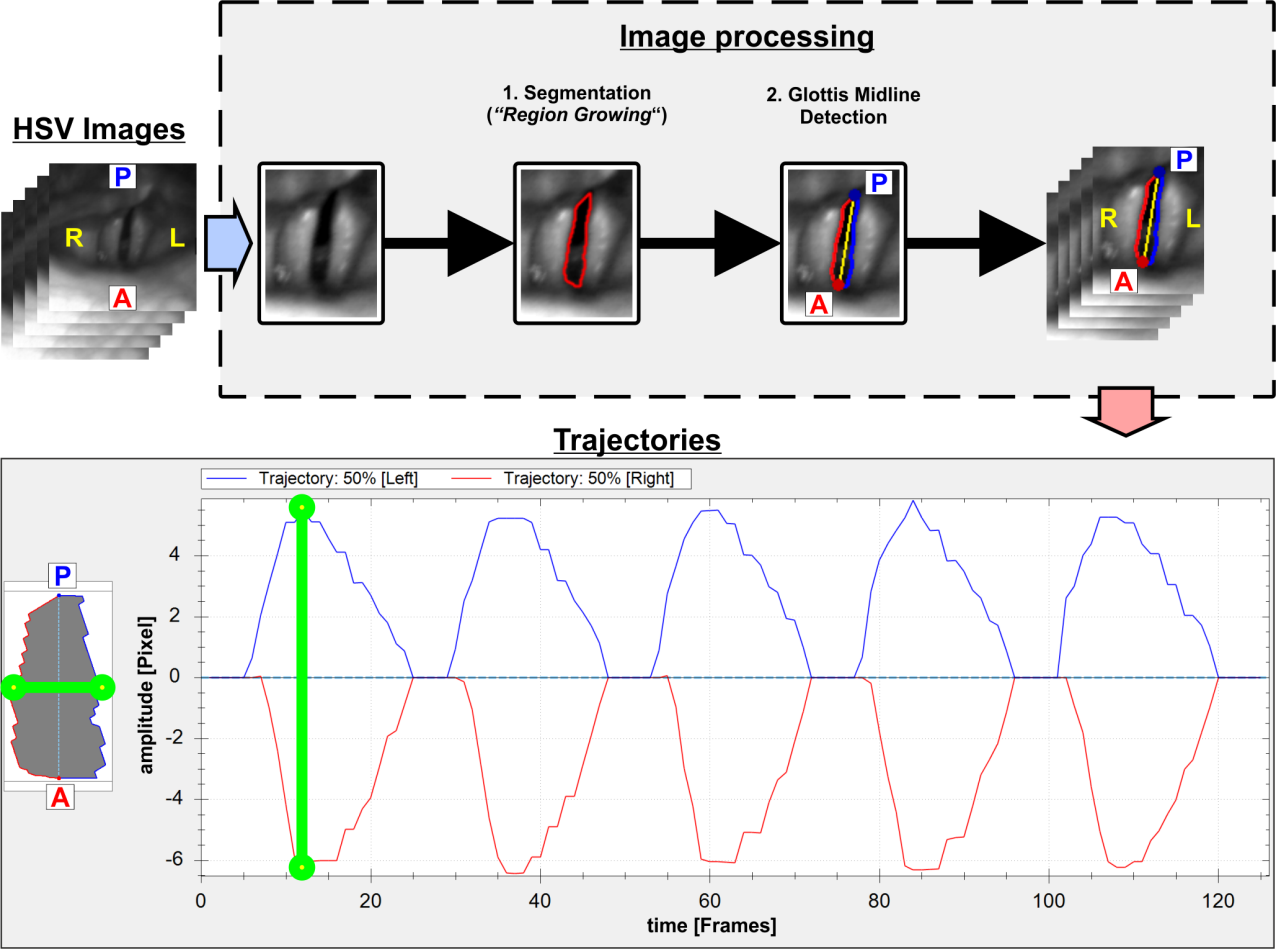
Performed steps for image processing yielding the experimental vocal fold trajectories.

First, image processing was performed to determine the glottis area and vocal fold edges [11], Fig 3. The glottis axis was determined using the methods being described in [71,72], Fig 3. The in-house developed software tool “Glottis Analysis Tool” (GAT) was used. The GAT tool has already proven its validity and applicability within several studies [8,73] and is also used by other voice groups [74,75].

For the adaptation of the 2MM, the trajectories from the mid-membranous position, 50% position between anterior and posterior, of the vocal folds were chosen (Fig 3), since the largest vocal fold amplitudes are expected in this region [76]. The trajectories at the medial vocal fold position are also expected to be the most useful [16]. To guarantee the extraction of the vocal fold trajectories at the 50% glottal mid-line only HSV recordings were used, where no obstructions of the view by the epiglottis was present and the most anterior and the visible parts of the posterior glottis were in view.

### Two-mass model (2MM)

**Fig 4:**
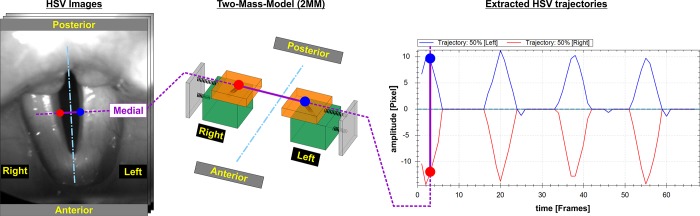
HSE image with indicated glottal axis (vertical blue line) and medial positions (~50% position between anterior and posterior) on left (blue dot) and right (red dot) vocal folds where the trajectories were extracted (left figure). The 2MM used (middle figure). Extracted trajectories for left (blue) and right (red) vocal folds (right figure).

The human voice is generated by three-dimensional vocal fold oscillations [76]. During phonation, oscillations of the vocal fold mucosa occur in the anterior–posterior, medio-lateral and vertical directions [20]. Anterior–posterior movements are fairly small and can therefore be neglected [76]. The vertical displacements (up to 2.4 mm) are approximately two-thirds of the dominant medio-lateral displacements [33,76]. However, the vertical component cannot be reconstructed based on the current HSV imaging techniques, owing to the lack of a second imaging tool (e.g., a second camera or laser projection system [77]). So far, extended studies on three-dimensional reconstruction of vocal fold surface dynamics have only been possible in ex-vivo or in synthetic experiments [77,78]. For three-dimensional in-vivo reconstruction, only case studies [79] and proof of concepts [80] were performed lately. Therefore, we focused on the dominant medio-lateral (i.e., horizontal) displacement characteristics at one vocal fold position that can be simulated by the 2MM (Fig 4).

Important laryngeal parameters that influence the oscillations are vibrating vocal fold masses (m), tension, elasticity, damping (r), stiffness (k) and subglottal air pressure (P_s_). The 2MM enables these parameters to be varied and the effects on the medio-lateral oscillatory behavior at one specific vocal fold location to be observed. Since the here applied 2MM has already been extensively described [28], we will give only information necessary to understand the functionality of the 2MM and the optimization procedure. The 2MM is based on [27,28] and assumes that each vocal fold is formed by two vertically arranged coupled masses: A larger, lower mass (m_1α_) and a smaller, upper mass (m_2α_). Each part consists of a simple mechanical oscillator with a mass and two springs at which the two masses of one vocal fold are connected by one spring. The 2MMs driving force is the subglottal pressure P_s_ below the masses. The 2MM is described by a system of eight (α = l,r) ordinary differential equations [25]:

$$\begin{array}{l} \frac{d}{dt}\left(\begin{array}{c} \begin{array}{c} x_{1\alpha }\\ v_{1\alpha } \end{array}\\ \begin{array}{c} x_{2\alpha }\\ v_{2\alpha } \end{array} \end{array}\right)=\left(\begin{array}{cccc} 0 & 1 & 0 & 0\\ -\left(\frac{k_{1\alpha }+k_{c\alpha }}{m_{1\alpha }}\right) & -\frac{r_{1\alpha }}{m_{1\alpha }} & \frac{k_{c\alpha }}{m_{1\alpha }} & 0\\ 0 & 0 & 0 & 1\\ \frac{k_{c\alpha }}{m_{2\alpha }} & 0 & -\left(\frac{k_{2\alpha }+k_{c\alpha }}{m_{2\alpha }}\right) & -\frac{r_{2\alpha }}{m_{2\alpha }} \end{array}\right)\left(\begin{array}{c} \begin{array}{c} x_{1\alpha }\\ v_{1\alpha } \end{array}\\ \begin{array}{c} x_{2\alpha }\\ v_{2\alpha } \end{array} \end{array}\right)\\ \;+\;\left(\begin{array}{c} \begin{array}{c} 0\\ I_{1\alpha }\left(x_{1l},x_{1r}\right)+\frac{1}{m_{1\alpha }}F_{1}\left(x_{1r},x_{2r},x_{1l},x_{2l}\right) \end{array}\\ 0\\ I_{2\alpha }\left(x_{2l},x_{2r}\right) \end{array}\right) \end{array}$$ (1)

The indices (i, α) represent lower (i = 1) and upper (i = 2) masses; α = l,r represents the left and right vocal fold. Ishizaka & Flanagan (1972) introduced a standard parameter value set that represented the standard vocal fold vibration pattern [81]. The parameters from Eq (1) and their standard values as originally introduced [28,81] are given in Table 1. The nonlinear components (I_1α_, I_2α_ and F_1_) describe the impact forces (I_1α_ I_2α_) and the subglottal pressure function (F_1_), described also in detail in earlier work [25].

**Table 1:** Standard parameters of the 2MM. In this study, the chosen vocal fold lengths l were 10 mm for women and 16 mm for men. The rest positions x_01_, x_02_ for the 2MM optimization were computed based on the mean amplitudes of the HSV trajectories yielding also individual rest areas a_0i_ [26]. During the 2MM optimization, the m_i_, k_i_ and P_s_ values are varied.

m_1_ [g]	m_2_ [g]	k_1_	k_2_	k_c_	r_1_	r_2_	c_1_
0.125	0.025	0.08	0.008	0.025	0.02	0.02	3k_1_
**c_2_**	**x_01_ [cm]**	**x_02_ [cm]**	**a_01_**	**a_02_**	**d_1_ [cm]**	**l [cm]**	**P_s_ [cmH_2_O]**
3k_2_	0.0179	0.0179	0.05	0.05	0.25	1.4	8.0

For the optimization, the 2MM trajectories (T_Mα_, α = l,r) from each side are a combination of the displacements of the lower and upper masses. The mass (m_1α_, m_2α_) that is closer to the glottal midline (i.e., visible from above) contributes to the corresponding vocal fold trajectory T_Mα_ [40].

The HSV recordings do not allow the extraction of the vocal fold oscillations in metric units but only in pixels. Owing to the known differences in vocal fold length, we chose mean values for the vibrating vocal fold length to be 10 mm in women and 16 mm in men. These values are the average membranous vocal fold lengths being reported in previous work [82–86]. Due to the metric mapping of the vocal fold lengths, we can calculate an approximate metric equivalent of the length of one pixel and convert the extracted trajectories to metric units.

During the optimization, the parameters vocal fold mass (m_1α_), stiffness (k_1α_), and subglottal pressure (P_s_) are varied. This is based on the study by Steinecke and Herzel (1995) [28], who also introduced a scaling factor Q_α_ (α = l,r) to vary the standard masses (m_1α0_) and spring variables (k_1αo_). Laryngeal asymmetry is expressed by the scaling factors Q_α_ The scaling factors Q_l_ (left vocal fold) and Q_r_ (right vocal fold) influence the masses and spring constants in the following way [27,28]:

$$\begin{array}{l} k_{i\alpha }=Q_{\alpha }k_{i\alpha 0}{,\;k}_{c\alpha }=Q_{\alpha }k_{c\alpha 0}\\ c_{i\alpha }=Q_{\alpha }c_{i\alpha 0},\;m_{i\alpha }=m_{i\alpha 0}/Q_{\alpha } \end{array}$$ (2)

This reciprocal relationship between vibrating masses m_1α_ and springs k_1α_ is based on the assumption that the larger the vibrating mass, the smaller is the stiffness of the vocal folds [28]. The 2MM oscillates symmetrically provided that Q_l_ and Q_r_ are equal or only slightly different. If the differences between the Q_i_ are too large, the 2MM vibrations become left-right asymmetric.

### Optimization procedure

**Fig 5:**
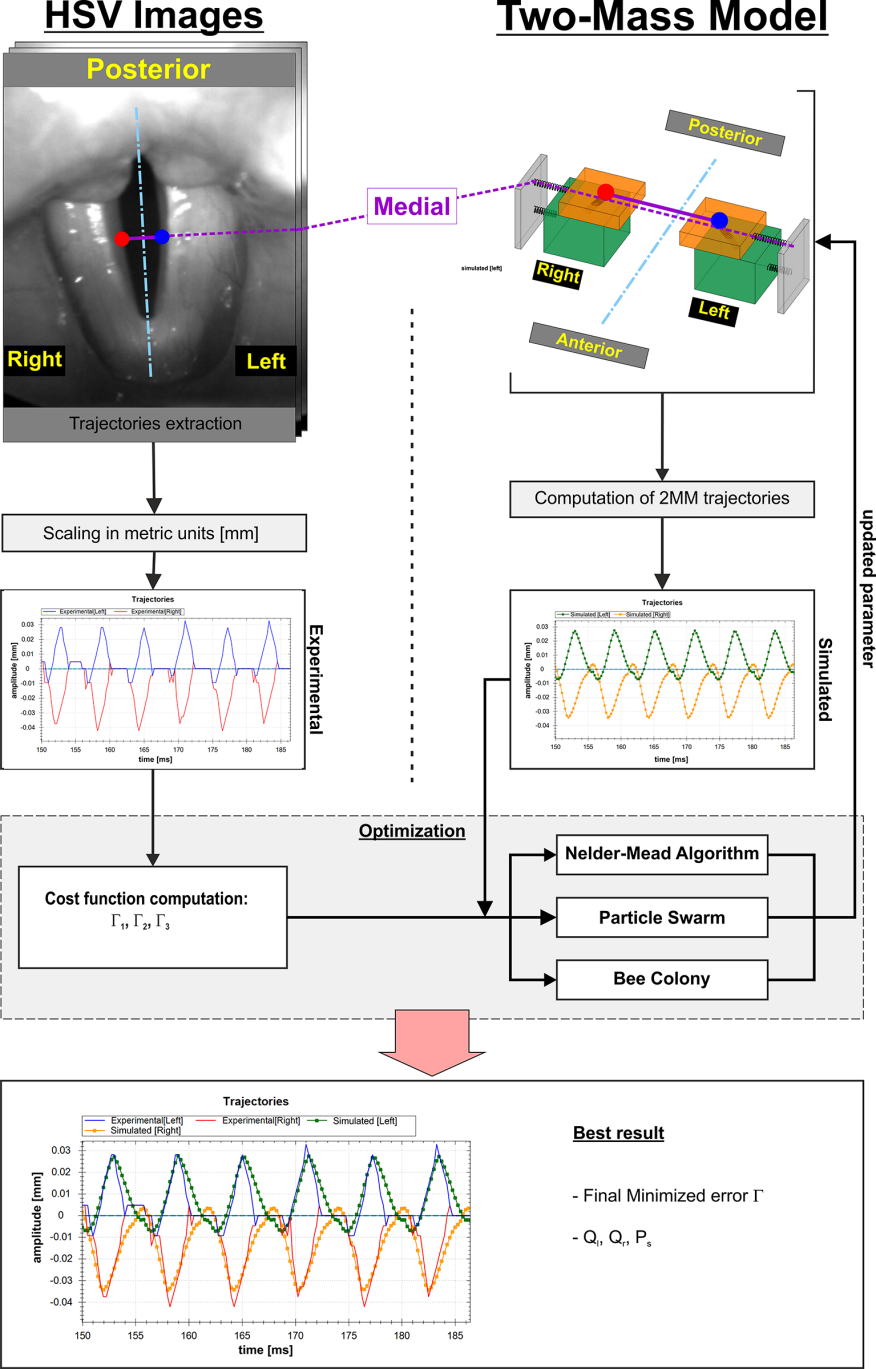
Flow diagram of the optimization procedure.

With the variation of Q_l_ and Q_r_ reflecting mass and stiffness for each vocal fold and the subglottal pressure P_s_, it is possible to reproduce physiologic and pathologic vocal fold oscillations [26,36]. The goal of the optimization is to vary these parameters so that the resulting 2MM trajectories (T_M_) accurately recreate the HSV recorded and extracted vocal fold trajectories (T_E_). This is realized by a combination of several steps within the optimization algorithm (Fig 5). The cost function judges the quality of the 2MM optimization and compares the model trajectories T_Ml_ and T_Mr_ with the recorded vocal fold trajectories T_El_. and T_Er_. Three different cost functions (Γ_1_, Γ_2_, Γ_3_), as described below, are used to match the extracted vocal fold trajectories as closely as possible.

#### Frequency domain (Γ_1_)

The absolute and phase values of the dominant harmonics between T_M_ and T_E_ are considered as suggested previously [25]. First, a preprocessing step with a Fourier transform for identification of the harmonics involved in the left experimental vocal fold trajectory (T_El_) and right trajectory (T_Er_) is performed. The experimental trajectory’s Fourier spectrum is dominated by only a small number of harmonics, represented by their Fourier coefficients e_iα_ (i = number of harmonic, α = left (l) or right (r)). Only harmonics e_iα_ that exhibit at least 25% of the absolute value of the largest coefficient e_1α_ = max (e_iα_) representing the fundamental frequency are taken into account. For irregular or aperiodic vibrations the coefficient e_1α_ = max (e_iα_) corresponds to the dominant frequency. Additionally, the next left and right neighbors of the coefficients are selected to consider slight variations in the harmonics [25]. For the simulated 2MM trajectories T_Ml_ and T_Mr_, the equivalent Fourier coefficients (s_jα_, i = j) are chosen and considered in the cost function Γ_1_:

$$\begin{array}{c} \Gamma_{1}\left(Q_{l},Q_{r},P_{s}\right)≔\\ s\cdot ∥\left(|e_{1l}|,\ldots ,|e_{Ll}|\right)-\left(|s_{1l}|,\ldots ,|s_{Ll}|\right){∥}_{2}\\ +∥\left(arg\;e_{1l},\ldots ,arge_{Ll}\right)-\left(args_{1l},\ldots ,args_{Ll}\right){∥}_{2}\\ +\;s\cdot ∥\left(|e_{1r}|,\ldots ,|e_{Rr}|\right)-\left(|s_{1r}|,\ldots ,|s_{Rr}|\right){∥}_{2}\\ +∥\left(arge_{1r},\ldots ,arge_{Rr}\right)-\left(args_{1r},\ldots ,args_{Rr}\right){∥}_{2} \end{array}$$ (3)

In Γ_1_, *L* corresponds to the number of coefficients for the left and *R* for the right vocal fold. Also, a scaling factor *s* is included in Γ_1_ to balance the influence of phase and absolute values of the Fourier coefficients as described in [25]. Γ_1_ was constructed to reduce the number of local minima and to therefore potentially yield improved optimization results [87].

#### Time domain (Γ_2_)

The Euclidean distance between the model trajectories (T_Ml_, T_Mr_) and the recorded trajectories (T_El_, T_Er_) is computed.

#### Normalized frequency domain (Γ_3_)

This cost function consists of Γ_1_, except that the absolute Discrete Fourier Transform (DFT) coefficients are normalized to 1 and that an additional regularization term is added: the Euclidean distance of the absolute values of the DFT coefficients representing the fundamental frequency.

#### Optimization was rated successful when the following three error criteria were achieved

(1) Frequency deviation ≤ 5%; (2) glottis closure as seen in the HSV recordings was achieved for the optimized 2MM trajectories; (3) amplitudes of optimized 2MM trajectories were within the amplitude variations of HSV trajectories; see e.g., Fig 4 where the right trajectory (red) varies between 12.5 and 15 pixel. Optimization would be defined successful for this trajectory when the corresponding right 2MM amplitude was also between 12.5 and 15 pixel.

For optimization, three algorithms are run separately on all three cost functions Γ_1_, Γ_2_, Γ_3_: (1) the Nelder–Mead (NM) algorithm [25], (2) the Particle Swarm Optimization (PSO) algorithm [45] and (3) the Simulated Bee Colony (SBC) optimization [88]. This yielded altogether nine optimized parameter sets (Q_l_, Q_r_, P_s_) approximating the recorded vocal fold trajectories T_Eα_. Since, the cost functions Γ_1_, Γ_2_, Γ_3_ are computed in different ways and in different domains (frequency and time) their absolute values cannot be compared to judge which cost function is actually better. Hence, the final and best parameter set (Q_l_, Q_r_, P_s_) was determined as the parameter set having the smallest normalized Euclidian distance Γ between the experimental T_Eα_ and simulated model trajectories T_Mα_:

$$\Gamma =\frac{1}{2}\left(\sqrt{\frac{\sum_{i=1}^{N}{\left(T_{El}[i]-T_{Ml}[i]\right)}^{2}}{\sum_{i=1}^{N}{\left(T_{El}[i]\right)}^{2}}}+\sqrt{\frac{\sum_{i=1}^{N}{\left(T_{Er}[i]-T_{Mr}[i]\right)}^{2}}{\sum_{i=1}^{N}{\left(T_{Er}[i]\right)}^{2}}}\right)$$ (4)

Prior to the actual optimization, for each subject an initial value search for Q_l_, Q_r_, and P_s_ was performed for the NM and PSO algorithms to reduce the potential search space and computational time for the actual optimization process [25]. An initial search was not performed for the stochastic based SBC algorithm.

The entire optimization process was performed on an Intel® Core™ i5-4590 Processor (3.30 GHz) using an in-house developed software written in C#. The software contained a GUI for improved handling and visually reviewing the results.

### Parameter analysis

For judging the left-right asymmetry in the 2MM and therefore in the vocal fold oscillations a factor Q_lr_ (≥ 1) is used, adapted from [28]. The closer the Q_lr_ quotient is to 1, the higher is the dynamic left–right symmetry:

$$Q_{lr}=\frac{max(Q_{l},Q_{r})}{min(Q_{l},Q_{r})}$$ (5)

Pairwise group differences (young females vs. young males) for the computed parameters (Q_l_, Q_r)_, Q_lr,_ P_s_ and Γ were statistically investigated. (Q_l_, Q_r_) are merged to one data pool, since the absolute differences between both the groups are of interest. Initially, to test for normal distribution, the Shapiro–Wilk test was used. All four parameters were not normally distributed: (Q_l_, Q_r_) (*df* = 50, *p* = 0.000), Γ (*df* = 25, *p* = 0.005), Q_lr_ (*df* = 25, *p* = 0.011) and P_s_ (*df* = 25, *p* = 0.001). Hence, Mann–Whitney U-tests were applied for the four group comparisons; the significance level was set to *p* = 0.05 and no Bonferroni correction was applied.

For comparing the younger vs. elderly subjects and gender specific differences in the elderly groups, only descriptive statistics were applied, due to the small number of elderly subjects. Hence, these observations are limited and have no statistical evidence. Statistical analysis was done using IBM SPSS Statistics 21.

## Results and discussion

### Applicability of the 2MM optimization

**Fig 6:**
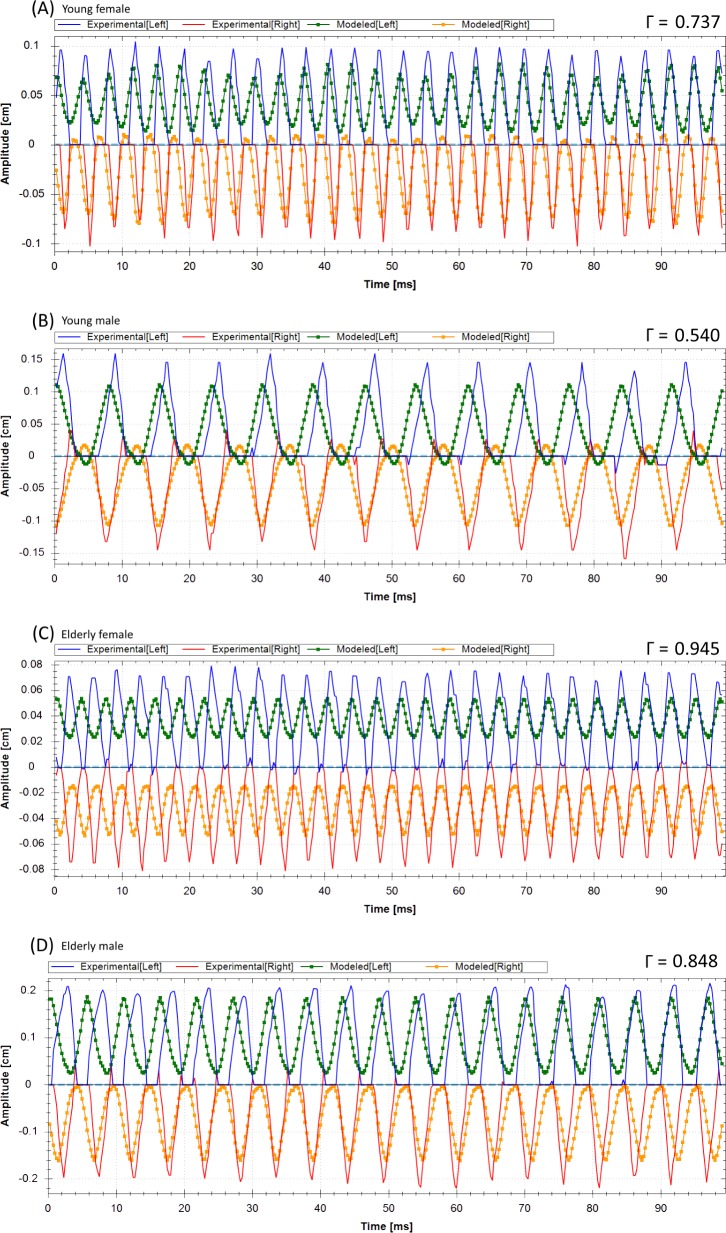
Examples for a young female (unmatched glottis closure and left amplitude), young male (unmatched amplitude), elderly female (unmatched glottis closure and amplitude) and elderly male (unmatched amplitudes and glottis closure) that illustrate the extracted trajectories and the incorrectly optimized trajectories of the 2MM for the left and vocal fold right side.

Results of the optimization procedure for the 2MM were only deemed correct, when all three above introduced error criteria were met. Altogether 12 HSV recordings (27.3%) could not be correctly optimized by violating one or more of these error criteria: (1) the fundamental frequency could not be matched (two times); (2) the HSV trajectories and the optimized 2MM amplitudes did not match (nine times); (3) the glottis closure or the glottis closure insufficiency was not reproduced (five times). In Fig 6, typical examples for failed optimization results are given: (A) For the young female the Γ value was within the range of the correct rated optimizations, however glottis closure was not achieved. (B) For the young male the Γ value is in the upper range of the correctly rated optimizations, however the amplitudes did not match. (C) For the elderly female the Γ value was higher than for the correctly rated optimizations, glottis closure was not achieved and the amplitudes did not match. (D) For the elderly male the Γ value was higher than for the correctly rated optimizations, glottis closure was not achieved and the amplitudes did not match.

Altogether 72.7% of the HSV trajectories were successfully optimized: 68.8% (11 out of 16) of the young females, 82.4% (14 out of 17) of the young males, 80.0% (4 out of 5) of the older females, and 50.0% (3 out of 6) of the older males. In the following, only these 32 successfully optimized HSV recordings will be considered and discussed.

As can be seen in Table 2, all three applied optimization algorithms yielded optimal results (i.e., lowest Γ value). However, the NM (38%) and SBC (43%) algorithms yielded more often the best results than the PSO (19%) algorithm. For the cost function, Γ_2_ definitely showed the most promising results. Γ_2_ yielded the best results most often (94%) followed by Γ_3_ (6%). Γ_1_ never yielded the best approximation. With regard to our first aim, the results suggest that all three optimization algorithms are suitable for the 2MM optimization but as cost function only Γ_2_ seems to be promising.

**Table 2:** Overview of how often each optimization algorithm (Nelder Mead–NM, Particle Swarm Optimization–PSO, Simulated Bee Colony—SBC) and cost function Γ_1_, Γ_2_, and Γ_3_ yielded the best optimization result; i.e., smallest Γ value–see Eq (4).

	Algorithms yielding best performance			Cost functions yielding best performance		
	NM	PSO	SBC	Γ_1_	Γ_2_	Γ_3_
**Young Males**	6	2	6	0	13	1
**Young Females**	4	2	5	0	10	1
**Older Males**	1	0	2	0	3	0
**Older Females**	1	2	1	0	4	0
**Sum**	12	6	14	0	30	2

The low values of the objective function Γ confirm the applicability of the 2MM for all four groups, Table 3. A value of Γ = 0 would correspond to a perfect optimization without any discrepancies between experimental and simulated curves. The highest mean Γ values are for elderly males (0.63 ± 0.09), followed by elderly women (0.59 ± 0.18). The best and lowest values are found for young men (0.45 ± 0.06) followed by the young women (0.57 ± 0.20). The difference in Γ (young women vs. young men) is not statistically different (*p* = 0.434).

**Table 3:** Mean values, standard deviations and range (minimum–maximum) of *Γ*, the optimized parameters P_s_ [cmH_2_O], Q_l_, Q_r_ and the symmetry quotient Q_lr_ for the four subject groups are given.

	Young Males		Young Females		Elderly Males		Elderly Females	
	Mean ± SD	Range	Mean ± SD	Range	Mean ± SD	Range	Mean ± SD	Range
*Γ*	0.45 ± 0.06	0.37–0.57	0.57 ± 0.20	0.32–0.90	0.63 ± 0.09	0.58–0.74	0.59 ± 0.18	0.40–0.82
**P**_**s**_ **[cmH**_**2**_**O]**	16.49 ± 7.13	10.10–32.31	21.12 ± 7.16	13.20–36.10	22.61 ± 6.50	15.14–27.00	28.30 ± 12.17	18.70–45.70
**Q**_**l**_	1.12 ± 0.32	0.76–1.99	2.61 ± 0.38	2.03–3.32	1.32 ± 0.15	1.19–1.49	2.81 ± 0.93	1.56–3.70
**Q**_**r**_	1.15 ± 0.28	0.78–1.83	2.46 ± 0.34	1.93–3.05	1.68 ± 0.62	1.15–2.36	3.23 ± 1.35	1.60–4.87
**Q**_**lr**_	1.07 ± 0.04	1.02–1.17	1.12 ± 0.08	1.01–1.24	1.32 ± 0.24	1.10–1.59	1.12 ± 0.13	1.02–1.32

The values suggest that the varied masses and stiffness parameters of the 2MM might adapt slightly better to the two younger subject groups than they do when elderly subjects are considered. However, higher values of Γ were expected for the elderly groups in comparison with young adults, as elderly subjects were reported to have lower laryngeal dynamic periodicity compared with younger adults [89,90]. This means that the glottis and therefore the extracted vocal fold trajectories oscillate not as periodically as they do in young adults. The 2MM does not allow for the simulation of slight changes in oscillation period length and slight amplitude changes between oscillation cycles (i.e., Jitter and Shimmer) hence yielding consequently higher Γ values for the elderly subjects.

**Fig 7:**
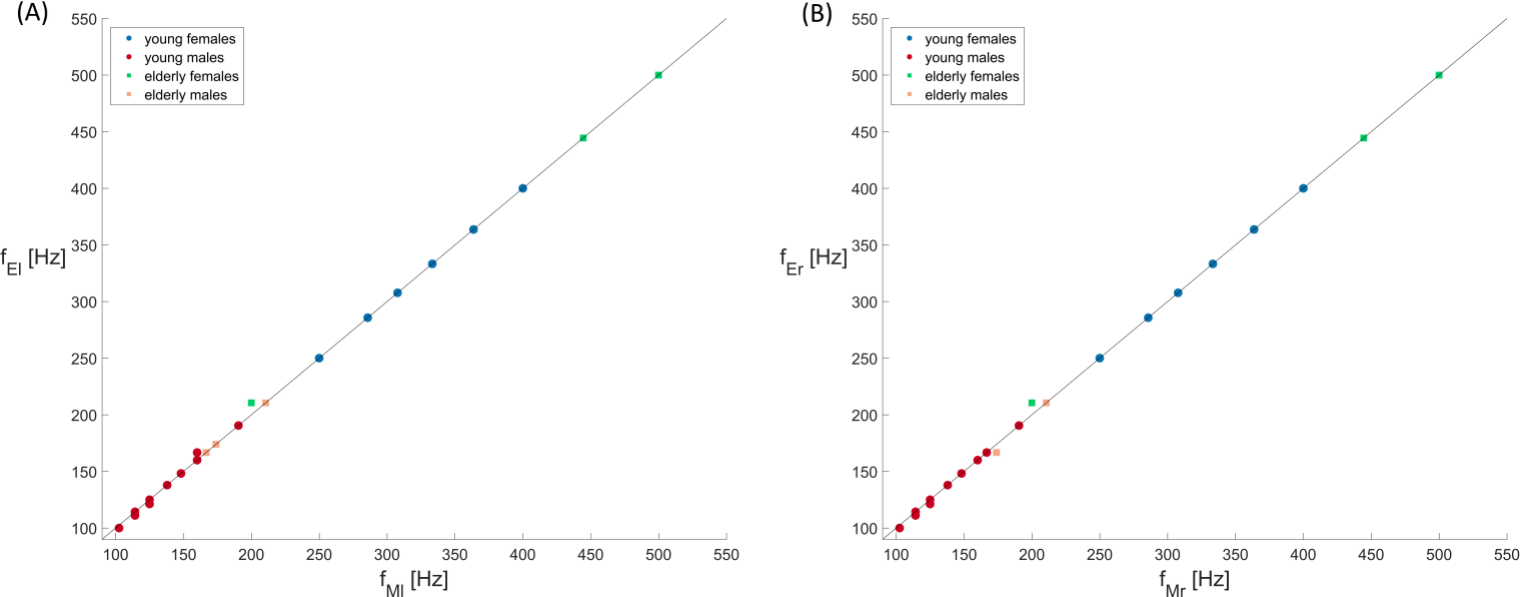
Fundamental frequencies (f_El_, f_Er_) of the experimental HSE-recorded trajectories versus the frequencies (f_Ml_, f_Mr_) of the optimized model trajectories shown separately for left and right vocal folds.

The accuracy of the optimization regarding the fundamental oscillation frequencies of the vocal folds is illustrated in Fig 7, where the experimental trajectory frequencies (f_El_, f_Er_) are plotted against the optimized 2MM frequencies (f_Ml_, f_Mr_). The highest accuracy is given for young women, where the model and experimental frequencies match for all subjects. For young males, the frequencies match perfectly for eight subjects. For three subjects the frequencies deviate for one vocal fold side with Δ = {6.7, 3.8, 3.8 Hz} and for three subject the frequencies deviate for both vocal folds with Δ = {3.8, 3.2, 2.6 Hz}. For older women, the frequencies match for three subjects. For one subject the frequencies deviate for both vocal folds with Δ = {10.5 Hz}. For older men, the frequencies match for two subjects. For one subject the frequencies deviate for one vocal fold with Δ = {7.2 Hz}.

**Fig 8:**
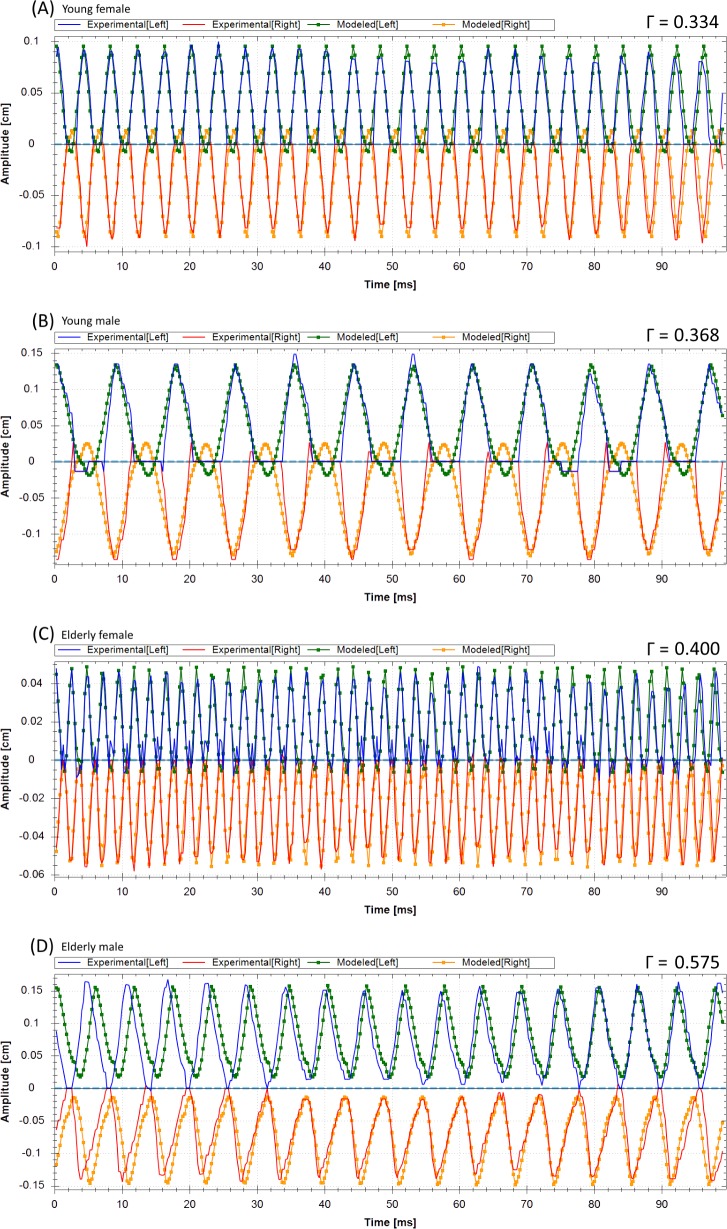
Examples for a young female, young male, elderly female and elderly male that illustrate the extracted trajectories and the correctly optimized trajectories of the 2MM for the left and vocal fold right side. The values for the cost function *Γ* are given.

In summary, 81% of the successful optimized trajectories *f*_*Mα*_ matched the fundamental frequencies of the experimental trajectories *f*_*Eα*_. This value is similar to that reported previously [26], where 80% of the original fundamental frequencies were correctly reproduced. Fig 8 shows examples for correctly reproduced vocal fold trajectories.

### Optimized parameters

The computed values for P_s_, Q_lr_, Q_l_ and Q_r_ and found group differences (gender and age) confirm our hypotheses as formulated in aims (2) and (3):

Table 3 gives an overview of the determined P_s_, Q_l_, Q_r_ and Q_lr_ parameter values. The **symmetry quotient Q**_**lr**_ shows high symmetry for the two young healthy groups, confirming the previously performed medical diagnosis of normal voice production. In our study, young women and men showed similar, statistically not significant differences with *p* = 0.202, and highest symmetry with Q_lr_ values of 1.07 ± 0.04 for young males. Young (Q_lr_ = 1.12 ± 0.08) and older females (Q_lr_ = 1.12 ± 0.13) exhibit equal symmetry. Deviations of dynamic left–right symmetry of up to 20% (i.e., Q_lr_ ≈ 1.20) were reported previously [42] and can still be considered as entirely physiologic. Further, slight physiologic and anatomic asymmetries were reported in healthy young and elderly subjects [69,70]. However, the older the subjects, the more prominent and larger the vocal fold dynamic asymmetries might become [91]. This was reflected only for the older male group (Q_lr_ = 1.28 ± 0.20) that showed increased asymmetry values (i.e., higher Q_lr_ values). The older female group was much more symmetric than the elderly male group. These findings confirm our hypothesis of higher kinematic asymmetries in elder subjects for women but not for men.

Young men showed the lowest **subglottal pressure P**_**s**_ with a mean value of 16.49 ± 7.13 cmH_2_O, but also had the lowest fundamental frequency (147 ± 38 Hz). In contrast, older males showed clearly higher subglottal pressure at 22.61 ± 6.50 cmH_2_O. Also the fundamental frequency for elderly men was increased (182 ± 22 Hz) confirming earlier studies [62]. Also, for the elderly females the fundamental frequencies (380 ± 117 Hz) were increased compared to young women (328 ± 40 Hz)—contradicting previous observations [92]. Also the subglottal pressures (28.30 ± 12.17 cmH_2_O) were higher for the elderly compared to the younger female group (21.12 ± 7.16 cmH_2_O).

The two elderly male and female groups showed both higher subglottal pressure and higher fundamental frequencies compared to their corresponding younger groups. The subglottal pressure for the male groups was smaller compared to the corresponding female groups. Comparing the young gender groups revealed statistically significant differences (*p* = 0.021); young men showed smaller P_s_ than young women. This is in contrast to previous studies where males and females showed similar values for both groups (Table 4). Overall, the computed subglottal pressures (10.10–45.70 cmH_2_O) were much higher compared to previously reported in-vivo value ranges (normal phonation: 3.5–12.8 cmH_2_O, loud phonation: 5.9–27.7 cmH_2_O), Table 4. However, the computed P_s_ values are in the same range as in previous studies (11.6 cmH_2_O ≤ P_s_ ≤ 46.3 cmH_2_O) that optimized the 2MM towards human in-vivo [26] and a 3DM model towards human ex-vivo [44] vocal fold dynamics. In [44], the computed P_s_ values very well approximated the applied and measured P_s_ values indicating that the here presented values may not be entirely off. High P_s_ values were also reported for human ex-vivo larynx experiments (up to 44.0 cmH_2_O in [96] and up to 35 cmH_2_0 in [97]). However, the computed P_s_ values in our study most likely overestimated the actual applied P_s_ values but are still in reported ranges.

**Table 4:** Overview on subglottal pressure vales (cmH_2_0) as reported for healthy subjects during normal and loud phonation in the literature.

Study	Females		Males	
	Normal	Loud	Normal	Loud
**Holmberg et al [93]**	3.6–8.1	6.4–13.7	4.3–9.7	6.2–16.4
**Perkell et al [55]**	5.5 ± 1.3	7.6 ± 1.8	5.9 ± 1.1	8.7 ± 2.4
**Sulter & Wit [54]**	11.2 ± 4.1	18.3 ± 6.9	11.8 ± 3.7	20.5 ± 5.9
**Baken & Orlikoff [94]**	3.5–12.6	6.5–27.7	4.2–12.8	5.9–24.4
**Hertegard et al [95]**	-	-	5–24	

**Q**_**l**_ **and Q**_**r**_ are investigated with regard to their absolute values. Clear differences between the four groups were apparent. Young men showed the lowest values for Q_l_ and Q_r_ (0.76–1.99), followed by the older males (1.15–2.36). The computed values are in the same range as reported previously [26]. Older women had the highest values (1.56–4.87) followed by young women (1.93–3.32). Transferring this to the vocal fold physiologically means that younger men and women have higher oscillating masses with smaller stiffness than their older comparison groups. Also, this means that men have larger masses and lower stiffness than the corresponding female groups. It has to be mentioned that the relation “increasing mass–decreasing stiffness” is induced by the modeling parameter Q_α_ as can be seen in Eq (2) [28]. However, it is generally understood that the vibrating portion of the vocal fold masses usually becomes smaller when vocal fold tension is increased [81, 98]. For young women vs. young men the difference for (Q_l_, Q_r_) was found statistically significant with *p* = 0.000.

**Fig 9:**
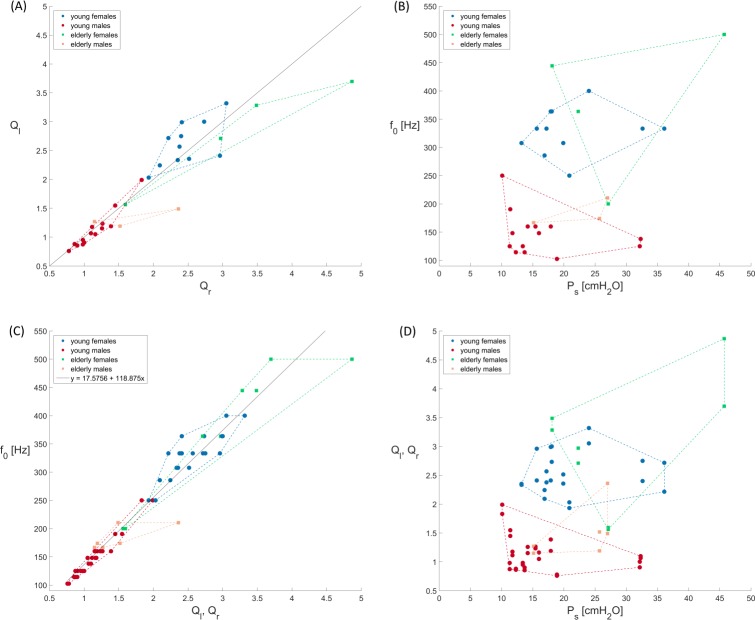
Scatterplots for the distribution of the four groups relating (A) Q_l_ vs. Q_r_ (B) the fundamental frequencies f_0_ vs. P_s_, (C) f_0_ vs. (Q_l_, Q_r_) and (D) (Q_l_, Q_r_) vs. P_s_.

For stiffness, the found gender differences confirm an earlier study where the amplitude quotient (AQ) as an indirect measure of the viscoelastic stiffness of vocal folds was used [15]. The amplitude quotient is determined by the shape and amplitude of the glottal area waveform. A smaller absolute value of amplitude quotient in young women in comparison with young males was reported, indicative of increased stiffness for the young females. However, it should be noted that the amplitude quotient is not an explicit measure of elasticity. Further, larger absolute values of maximum area declination rate in young women compared to young men were reported [15]. This is indicative of larger absolute peak velocity during the closing phase in young women, hinting to increased stiffness in young women and being again confirmed by the computed larger Q_l_, Q_r_ values, Fig 9A.

### Relationships between parameters

It is notable that the computed lower P_s_ values (10–20 cmH_2_O, being 28% of the entire P_s_ range) account for 97% of all occurring fundamental frequencies (100 Hz– 500 Hz), Fig 9B. This suggests that the subglottal pressure might play a minor role in frequency changes, as observed before [99]. Hsiao et al (2001) showed that the relationship between fundamental frequency and subglottal pressure depends on the tension of the larynx [99]. This means for our results that a lower tension or stiffness (small *Q*_*α*_), as computed for young and elderly males, also means lower fundamental frequencies compared to young and elderly females, as confirmed in Fig 9C, whereas in contrast the P_s_ values were only slightly reduced (see means in Table 3) and almost in the same range (Fig 9B). In contrast, higher tension, as computed for both female groups, presents higher fundamental frequencies (Fig 9C) at only slightly increased P_s_ values. The high dependency between f_0_ and stiffness is also expressed by a high Pearson correlation coefficient of 0.986 (*p* = 0.000). This relationship was also seen before when for a male and female group different loudness levels (soft–normal–loud: i.e., increasing stiffness) were analyzed [93]. However, this study reported slightly lower P_s_ values for women compared to men. In summary, the computed P_s_ values in our study (Table 3) and also the values presented by [93] do overlap for different analyzed subject groups and tasks showing a high inter-individual variability for P_s_.

Fig 9D shows the relationship of absolute stiffness and vibrating masses (Q_l_, Q_r_) to the subglottal pressure P_s_. Young males are clearly separated from young and old females. Older males slightly overlap with both female groups. Further, the Fig 9D shows that the values for both young groups are more centered whereas the values for both elderly groups are more spread out and seem not to be as consistent.

### Study limitations and outlook

This study has clear limitations due to the sample size. When comparing the optimized 2MM parameters statistical tests were only performed when comparing young men vs. young women. When comparing age related differences and elderly men vs. elderly women no statistical tests were performed and only non-statistical tested trends were described. This lack of statistical significance is clearly a major limitation. Also only healthy young and atrophic elderly subjects were considered. However, the study yielded clear trends and initial group data for younger healthy and elderly atrophic subjects. Future studies should also investigate how the study parameters vary in elderly and young adults during modified phonation (e.g., pitch raise [40]).

#### Model and optimization limitations

The applied 2MM allows simulation of vocal fold oscillations only in the medio-lateral and not in the vertical direction, as reported before for a three-dimensional model [44]. Also, vibrational characteristics and changes along the vocal fold length (anterior-posterior) cannot be captured by the 2MM since only the trajectories at mid-membranous position (50% of the vocal fold length) are simulated. Hence, anterior-posterior phase differences [100] and typical posterior gaps for female phonation [69] are not captured. For analyzing these characteristics the 6-Mass-Model should be applied and optimized [41]. However, investigating such phenomena was not the focus of this study and will be taken into account in our future work. Also the considered trajectories were always extracted at the standardized 50% vocal fold length position with the assumed largest amplitudes. However, the largest amplitudes vary around this position (from posterior to anterior: females (41.1% ± 10.8%) and males (46.5% ± 18.0%)) as reported in [16]. Hence in further studies the influence of this assumption and the potential discrepancy towards the exact individual largest amplitude should be investigated for the optimization results.

The computed and optimized P_s_ values seem to be overestimated by the 2MM since the found values are much higher than assumed and reported for in-vivo measurements (see Table 4), although such high P_s_ values were reported for ex-vivo studies. However, this issue has to be clarified in future work. In this context, it has to be noted that the goal of investigating and optimizing LMMs towards vocal fold dynamics is not to directly transfer the quantities of computed masses, stiffness and subglottal pressure but to uncover underlying biomechanical differences between vocal fold dynamics [26,28,81,101].

No subgradient-based algorithms were applied for the optimization [102]. Applying such algorithms may enhance the number of correct optimization results.

The success of the optimization procedure was assessed by three objective criteria. However, an explicit objective measure of how the shape of the trajectories was reproduced is a question for future studies.

A dependent variation of masses and stiffness parameters as initially suggested was performed [28]. However, the independent variation and optimization of masses and stiffness within the 2MM should be considered, since otherwise an increase of mass always goes along with a reduction of stiffness. This dependency might reduce the applicability to certain vocal fold oscillations and also might not reflect certain biomechanical constellations within the vocal folds. Time-dependent parameters should be taken into account, since the 2MM with time-independent parameters does not allow for entirely correct simulation of inter-cycle changes as seen in Fig 8D; i.e., vocal folds show closure during a few cycles and then they do not.

For future classification purposes (i.e., normal vs. pathologies), it might be interesting to vary additional biomechanical parameters like collision and contact forces [103], frequency dependent stiffness [104] and glottal flow [105].

Finally, to enable the clinical application of LMMs in the daily clinical routine in the future, the computational time has to be reduced. Currently, the optimization including the initial value search for one HSV recording takes approximately 60 minutes on a desktop computer.

#### HSV imaging limitations

HSV imaging projects the three-dimensional vocal fold vibrations and surfaces onto two-dimensional pictures and movies. The image processing detects the dark region between the two vocal folds as glottis. The positions of the most medial edges of the vocal fold tissue from image processing are taken as experimental trajectories (*T*_*Eα*_). The trajectories (*T*_*Mα*_) of the 2MM are built from the positions of the upper and lower mass (*m*_*α*_) depending on which mass is more medial; i.e., visible from above. Since the exact vertical positions of the trajectories within the HSV images cannot be determined, it is unclear if the vertical position (upper or lower mass) of the model trajectories actually corresponds to the same vertical region (superior or inferior vocal fold edge) of the extracted trajectories. Applying HSV in combination with a laser projection unit that allows the reconstruction of the three-dimensional positions of the entire visible vocal fold surfaces would solve this shortcoming [80,106]. The use of a laser projection unit with HSV would further allow for the extraction of the vertical trajectory components of the vocal folds (96); however then a more complex three-dimensional LMM will have to be applied for optimization; e.g. [34].

Owing to the lack of metric specifications in the HSV recordings, average vocal fold lengths were used for males and females, whereas the individual length of the vocal folds was not taken into consideration. Because of the absence of metric units, the recorded vocal fold trajectories are initially scaled in pixels and then transferred to metric units using the averaged vocal fold length. Hence, the amplitudes may not match the actual oscillation quantities accurately. This shortcoming will also be solved in future studies when using HSV in combination with a laser projection unit [106] allowing for the extraction of metric units for vocal fold trajectories and using individual vocal fold lengths in the optimization procedure.

## Conclusion

This study is the first approach to use a LMM for comparing age and gender related differences based on vocal fold dynamics recorded with endoscopic high-speed imaging. The parameter optimization objectively quantified biomechanical differences in terms of dynamic symmetry, subglottal pressure, vocal fold masses and stiffness, across gender and age. The results show promising findings for quantifying vocal fold dynamics and for differentiating normal from disordered voice as well as in differentiating between vocal fold pathologies. However, the 2MM does not have one-to-one correspondence to the actual values of the vocal fold masses, stiffness, and subglottal pressure, but allows for objectively evaluating the biomechanical interrelationships between these variables.

Three different optimization algorithms were tested including three different cost functions. For future studies, the results do not favor a specific optimization algorithm but clearly show that the Euclidian Distance of the trajectories (Γ_2_) should be chosen as cost function to achieve best results.

## Supporting information

S1 Data Individual successful and failed optimization results. (XLSX)
